# Integrated Analysis of miRNA-mRNA Expression in Mink Lung Epithelial Cells Infected With Canine Distemper Virus

**DOI:** 10.3389/fvets.2022.897740

**Published:** 2022-05-31

**Authors:** Qiang Chen, Mingwei Tong, Na Sun, Yong Yang, Yuening Cheng, Li Yi, Gaili Wang, Zhigang Cao, Quan Zhao, Shipeng Cheng

**Affiliations:** ^1^College of Veterinary Medicine, College of Animal Science and Technology, Jilin Agricultural University, Changchun, China; ^2^Key Laboratory of Special Animal Epidemic Disease, Ministry of Agriculture, Institute of Special Economic Animals and Plants, Chinese Academy of Agricultural Sciences, Changchun, China; ^3^College of Landscape Architecture, Changchun University, Changchun, China; ^4^School of Basic Medical Sciences, Shanxi Medical University, Taiyuan, China; ^5^College of Animal Science and Technology, Jilin Agricultural Science and Technology University, Jilin City, China; ^6^Jilin Academy of Animal Husbandry and Veterinary Medicine, Changchun, China

**Keywords:** canine distemper, Mv. l. Lu cells, miRNA, transcriptome, correlation analysis

## Abstract

Canine distemper (CD) caused by canine distemper virus (CDV) is one of the major infectious diseases in minks, bringing serious economic losses to the mink breeding industry. By an integrated analysis of microRNA (miRNA)-messenger RNA (mRNA), the present study analyzed the changes in the mink transcriptome upon CDV infection in mink lung epithelial cells (Mv. l. Lu cells) for the first time. A total of 4,734 differentially expressed mRNAs (2,691 upregulated and 2,043 downregulated) with |log_2_(FoldChange) |>1 and P-adj<0.05 and 181 differentially expressed miRNAs (152 upregulated and 29 downregulated) with |log_2_(FoldChange) |>2 and P-adj<0.05 were identified. Gene Ontology (GO) enrichment indicated that differentially expressed genes (DEGs) were associated with various biological processes and molecular function, such as response to stimulus, cell communication, signaling, cytokine activity, transmembrane signaling receptor activity and signaling receptor activity. Kyoto Encyclopedia of Genes and Genomes (KEGG) pathway enrichment analysis of the combination of miRNA and mRNA was done for immune and inflammatory responses, such as Janus kinase (JAK)-signal transducer and activator (STAT) signaling pathway and nuclear factor (NF)-kappa B signaling pathway. The enrichment analysis of target mRNA of differentially expressed miRNA revealed that mir-140-5p and mir-378-12 targeted corresponding genes to regulate NF-kappa B signaling pathway. JAK-STAT signaling pathway could be modulated by mir-425-2, mir-139-4, mir-140-6, mir-145-3, mir-140-5p and mir-204-2. This study compared the influence of miRNA-mRNA expression in Mv. l. Lu cells before and after CDV infection by integrated analysis of miRNA-mRNA and analyzed the complex network interaction between virus and host cells. The results can help understand the molecular mechanism of the natural immune response induced by CDV infection in host cells.

## Introduction

Canine distemper virus (CDV) is a small, negative-sense, membrane-encased, single-stranded RNA virus, belonging to the genus *Morbillivirus* and family Paramyxoviridae (1). Canine distemper (CD) was first reported by Jeneer in 1809, and CDV was first isolated and identified in the UK by Carre in 1905 (2). CDV can cause respiratory, digestive and nervous system diseases in animals, and its clinical manifestations are characterized by typical bipolar fever, upper respiratory tract inflammation, catarrhal pneumonia, gastroenteritis and non-suppurative encephalomyelitis (3). The main infectious sources of CDV are infected animals and their excreta, and the viral load in the respiratory tract, eye and nose secretions, excreta of infected animals. The main mode of transmission of CD is direct or indirect contact. CDV mainly infects animals through the respiratory tract and digestive tract. Dust and droplets containing virus particles are also important routes of virus transmission (4, 5). CD is an acute disease with high mortality and a wide range of hosts. However, most studies focused on the epidemiology of CDV, and there are few studies on its pathogenesis, especially for CDV infected lung epithelial cells.

Transcriptomics is a discipline that systematically studies gene transcription maps from the whole transcription level to reveal molecular regulatory networks and biological regulatory pathways. Transcriptomics, proteomics, metabolomics and other omics have been used as important tools in molecular research, among which transcriptomics has been widely used (6). As a bridge between DNA and protein, RNA plays an important role in genome regulation of life activities in living bodies (7). Therefore, it is necessary to explore and study RNA through transcriptome analysis. Several studies showed that transcriptome assembly identified a key molecular mechanism in host-pathogen interaction at the cellular level with Sapovirus-3 (SAV-3) infected macrophage, CDV-11 infected dog renal malignant histiocytosis cell and canine parvovirus (CPV) infected madin-darby canine kidney cells (8–11). MicroRNA (miRNA) is a kind of endogenous single-stranded non-coding small RNA with a length of 18–25 nucleotides, which is widely found in animals, plants and viruses. As a class of non-coding RNA, miRNA is independent in its transcription process and participate in the regulation of gene expression. It inhibits the degradation or translation of messenger RNA (mRNA) and affects protein-coding genes and expression (12, 13). miRNA plays an important role in various physiological processes of the body, such as cell proliferation and differentiation, signal transduction, energy metabolism, disease resistance and immune regulation (14, 15). Studies found that miRNAs could regulate a variety of immune functions and infectious diseases (16, 17). In addition, miRNA profile can be changed after infection with human immunodeficiency virus, varicella-zoster virus, hepatitis B virus and hepatitis C virus (18–21). However, transcriptome and miRNA studies on mink CDV are still insufficient.

In this study, we focused on global miRNA and mRNA expression profiling in mink lung epithelial cells (Mv. l. Lu) cells infected with CDV and explored the molecular regulatory pathways of miRNAs with common expression patterns, which may be involved in the CDV infection process. The findings of this study will provide new ideas for the possible host-meditated innate and protective antiviral immune responses.

## Materials and Methods

### Cell Culture and Viral Infection

Mv. l. Lu cells in this study were maintained in Minimal Essential Media (MEM, Gibco, Life Technologies, Grand Island, NY, USA) supplemented with 10% fetal bovine serum (FBS) (Invitrogen, Waltham, MA, USA) and cultured at 37°C in a 5% CO_2_ incubator. CDV PS strain (GenBank No.: JN896331) was isolated from dogs of CD in 2013 by our laboratory (22). Due to the low viral titer of PS strain and the presence of cytokines in the virus venom, the virus was proliferated by Mv. l. Lu cells and then collected. The virus was concentrated by PEG 6000 (Thermo Fisher Scientific, Waltham, MA, USA) and purified by super centrifugation using the sucrose gradient method (23). The purified viruses were stored at −80°C before use. The titer of the virus was determined by the median tissue culture infective dose method (24).

### Preparation of Cell Samples and RNA Isolation

The Mv. l. Lu cells were cultured and inoculated in cell culture vials containing about 10^7^ cells. When the cells' fusion degree reached approximately 70–80%, the CDV which were concentrated with PEG 6000 and purified by supercentrifugation using sucrose gradient method was inoculated with a multiplicity of infection (MOI) of 2. Uninfected Mv. l. Lu cells were used as controls. Infected cells and controls were then cultured in 5% CO_2_ at 37°C for 24 h. Subsequently, the suspensions were removed from each cell culture vial, and the cells were washed twice with pre-cooled phosphate-buffered saline (PBS) (Thermo Fisher Scientific, Waltham, MA, USA). The cells were fully lysed by percussing with pre-cooled PBS, and the lysate was transferred to a 1.5 mL enzyme-free tube to extract RNA.

The total RNA was isolated using TRIzol Reagent (Invitrogen, Waltham, MA, USA) according to the manufacturer's instructions and quantified by the NanoDrop ND-2000 (Thermo Fisher Scientific, Waltham, MA, USA) and an Agilent Bioanalyzer 2100 (Agilent Technologies, Santa Clara, CA, USA) to assess RNA quality. The total RNA was stored in −80°C for purifying the mRNAs and constructing small RNA libraries.

### Library Construction, mRNA Sequencing and Expression Profiling

After extraction of total cell RNA, magnetic beads with oligo-dT were used to combine the poly-A of the mRNA for purifying the mRNAs (25). The mRNAs were then mixed with fragmentation buffer (Thermo Fisher Scientific, Waltham, MA, USA) to obtain short fragments of 200–300 bp. The fragments were used to synthesize first-strand complementary DNA (cDNA) with random primers, and first-strand cDNA was transformed into double-strand cDNA using RNase H and DNA polymerase I (Thermo Fisher Scientific, Waltham, MA, USA). Fragments of desirable lengths (200–300 bp) were purified using the QIA quick PCR Extraction Kit (Qiagen, Valencia, CA, USA). Under the function of 3′-5′ exonuclease and polymerase, the protruding terminus of the DNA fragments was end-repaired. Base “A” was induced at the 3′ end of DNA fragments. So, the base “T” at 3′ end of the adapters could complement the induced base “A.” The end-repaired DNA fragments were ligated with sequencing adapters through A and T complementary base pairing. AMPure XP beads (Beckman Coulter, Shanghai, China) were used to remove unsuitable fragments (<100 bp). Six sequencing libraries were constructed using polymerase chain reaction (PCR). The multiplexed cDNA libraries were checked using PicoGreen (Quantifluor^TM^-ST fluorometerE6090, Promega, Madison, WI, USA) and fluorospectrophotometry (Quant-iT PicoGreen dsDNA Assay Kit; Invitrogen, P7589, Waltham, MA, USA) and quantified with Agilent 2100 (Agilent 2100 Bioanalyzer, Agilent, 2100; Agilent High Sensitivity DNA Kit, Agilent Technologies, Santa Clara, CA, USA). The synthesized cDNA libraries were normalized to a 2 nM. The sequencing library was diluted and quantified to 4–5 pM and sequenced using the next-generation sequencing (NGS) and Illumina HiSeq platform.

The raw data were processed through the single base quality of sequencing data, base content distribution, GC content distribution and sequence base quality using FastQC (http://www.bioinformatics.babraham.ac.uk/projects/fastqc). Cutadapt (Version 1.2.1) in R language removed the connector sequence at the 3′ end and the low-quality reads to obtain clean reads. Clean reads were spliced by Trinity software (version r20140717, K-mer 25bp) in R language to obtain transcripts. The unigenes obtained by clustering were annotated for gene function using the National Center for Biotechnology Information (NCBI) non-redundant protein sequences (NR, http://www.ncbi.nlm.nih.gov/protein), Gene Ontology (GO, http://geneontology.org/) and Kyoto Encyclopedia of Genes and Genome (KEGG, http://www.kegg.jp/). DESeq (Version 1.18.0) in R language was used to analyze the differentially expressed unigenes, the screening criteria were |log_2_(FoldChange) |>1 and P-adj<0.05 (26). The volcano plot of differentially expressed unigenes was plotted by ggplot2 software package in R language. The hierarchical clustering analysis was performed to assess the expression profiles of the differentially expressed mRNAs by the Pheatmap software package of R language (https://cran.r-project.org/web/packages/pheatmap/index.html) and the Euclidean method.

### miRNA Expression Profiling

To identify changes in miRNA expression in Mv. l. Lu cells infected with CDV, two groups were assayed, including CDV infected group and uninfected group. Six miRNA libraries were constructed using TruSeq Small RNA Sample Prep Kit (Illumina, San Diego, CA, USA). The RNA samples were the same as the ones used for mRNA purification. The enriched libraries were amplified by PCR, and sequenced connector and index part were added. The libraries were quantified with Agilent 2100 (Agilent 2100 Bioanalyzer, Agilent, 2100; Agilent High Sensitivity DNA Kit, Agilent, Technologies, Santa Clara, CA, USA) and fluorospectrophotometry (Quant-iT PicoGreen dsDNA Assay Kit, Invitrogen, P7589, Waltham, MA, USA). Bridge PCR was performed with a single-chain library template, and the libraries were sequenced using the Illumina HiSeq platform.

The raw data were required to remove the joint and the quality of shearing, and the sequence was filtered for subsequent analysis. The expression data of conservative miRNA which screened by blast method to compare with animal mature miRNA sequences in miRBase 21 (http://www.mirbase.org/) sorted out, and the differentially expressed miRNAs were analyzed by DESeq (version 1.18.0) in R language. The differentially expressed miRNAs were identified according to the multiple difference (|log_2_(FoldChange) |>2) and significance of expression difference (P-adj < 0.05). GO (http://geneontology.org/) enrichment and KEGG (http://www.kegg.jp/) pathway analyses were done to determine the roles of 263,203 target genes.

### Correlation Analysis of miRNAs and mRNAs

To build a comprehensive miRNA-mRNA network with positive and negative correlations, the differentially expressed miRNAs and differentially expressed mRNAs with the opposite expression trend were selected. The miRanda (http://www.microrna.org/microrna/home.do), TargetScan (http://www.targetscan.org/) and DAVID (http://david.ncifcrf.gov/) were used to confirm the correlation of miRNA and mRNA. The differential miRNA-mRNA regulatory pairs were obtained with a score value > 170 and energy < −30, and Cytoscape version 3.0.1 (http://www.cytoscape.org/) in R language was used to construct regulatory networks. The annotations and enrichment of GO and KEGG were performed for differential miRNA corresponding to the different mRNAs obtained by combined analysis.

## Results

### mRNA Expression Profiling

**Figure 1:**
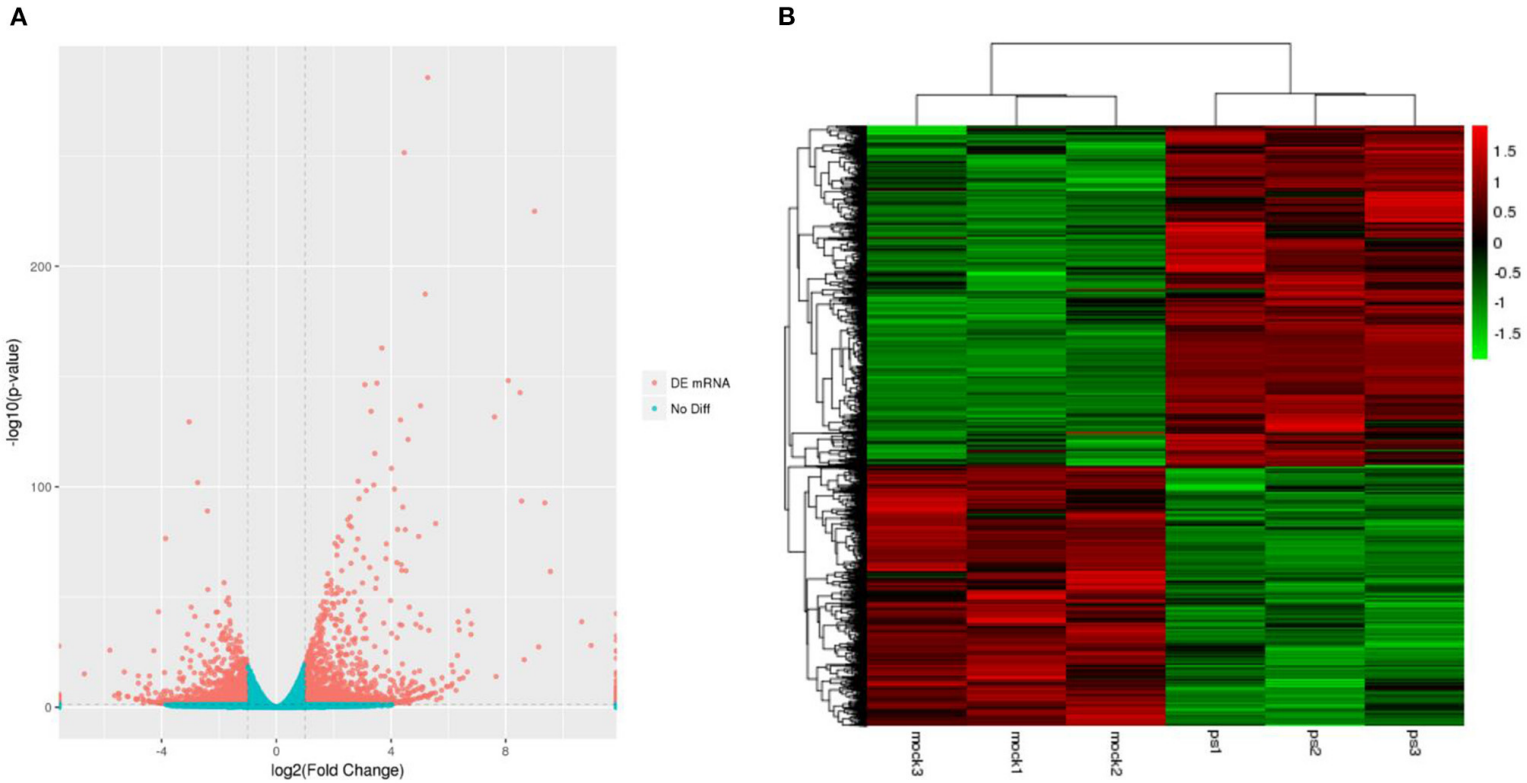
The expression profiling of mRNAs in the CDV-infected and control groups. **(A)** The volcano plot displaying the distribution of differentially expressed genes in infected groups compared with the uninfected group. Red dots indicate significantly upregulated genes and blue dot is no significantly different mRNAs. **(B)** Heat map showing the hierarchical clustering of mRNAs that are common to the six samples. Red and green indicate higher and lower expression in Mv. l. Lu cells, respectively. The color scale was the normalized count.

Six libraries for high throughput sequencing of mRNAs from the different groups were constructed, and these data were analyzed by RNA sequencing (RNA-Seq). The volcano plot of differentially expressed unigenes was showed in Figure 1A. Compared with the uninfected group, Mv. l. Lu cells infected with the CDV exhibited 4,734 differentially expressed unigenes (2,691 upregulated and 2,043 downregulated). The results of hierarchical clustering analysis were shown in a heatmap (Figure 1B). The mRNA expression profiles had significant differences between the virus infected and control groups.

### miRNA Expression Profiling

**Figure 2:**
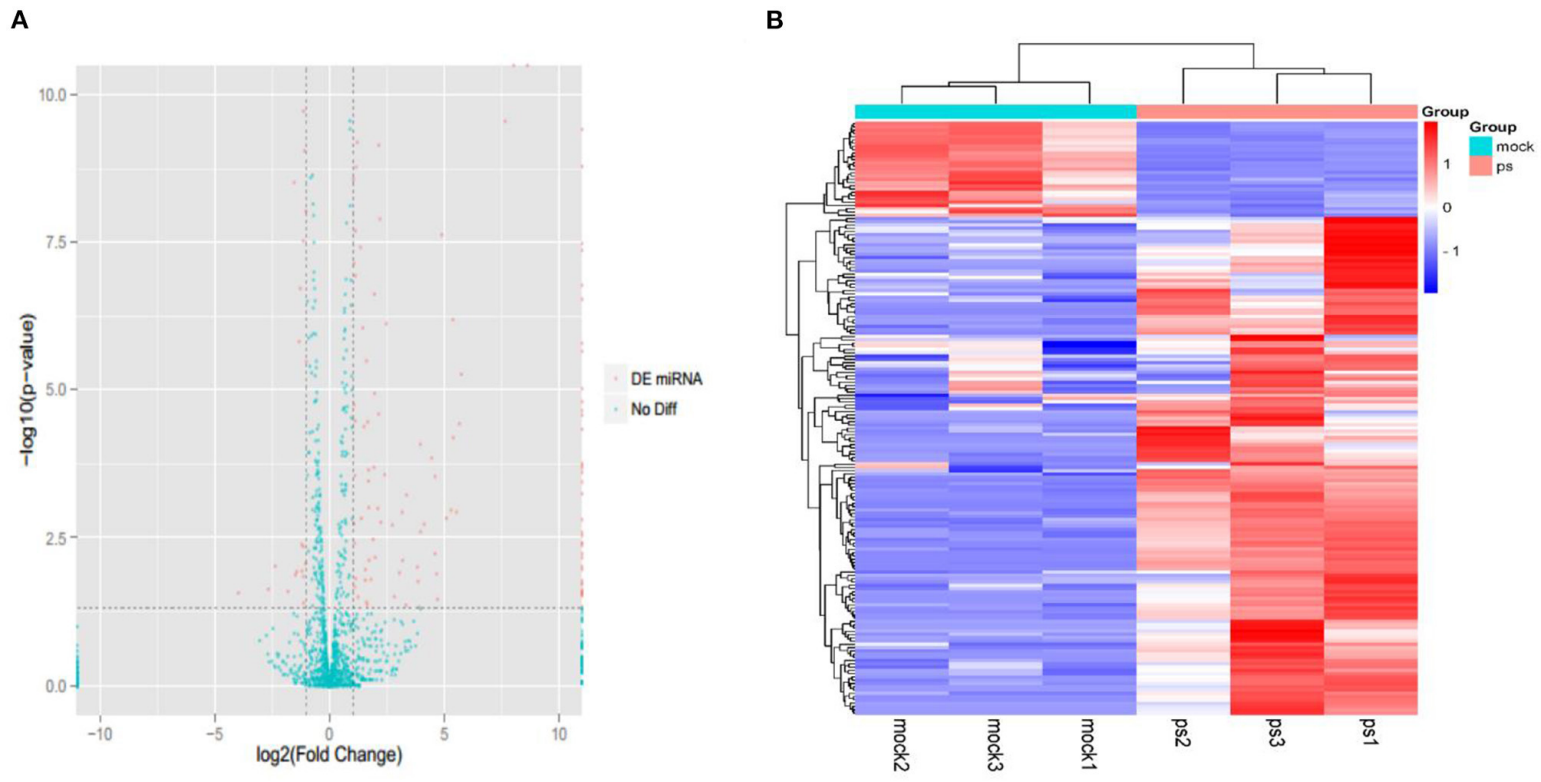
The expression profiling of miRNAs in the CDV-infected and control groups. **(A)** The volcano plot displaying the distribution of differentially expressed miRNAs in infected groups compared with the uninfected group. Red dots indicate significantly different miRNA and green dot is no significantly different miRNAs. **(B)** Heat map showing the hierarchical clustering of miRNAs that are common to the six samples. Red and purple indicate higher and lower expression in Mv. l. Lu cells, respectively. The color scale was the normalized count.

A total of six small RNA libraries were constructed, and each group was assayed in triplicate. The differentially expressed miRNAs were screened and displayed using volcano plots (Figure 2A), which showed that 181 differentially expressed miRNA were detected in the infected group compared with the uninfected group (152 upregulated and 29 downregulated). The upregulated miRNAs were detected more than downregulated miRNAs in the infected groups, indicating that the miRNAs in Mv. l. Lu cells were primarily upregulated after CDV infection. Furthermore, hierarchical clustering analysis (HCA) was used for expression profiling of the differentially expressed miRNAs to the different groups (Figure 2B).

### Integrated Analysis of miRNA and mRNA Expression Profiles

**Figure 3:**
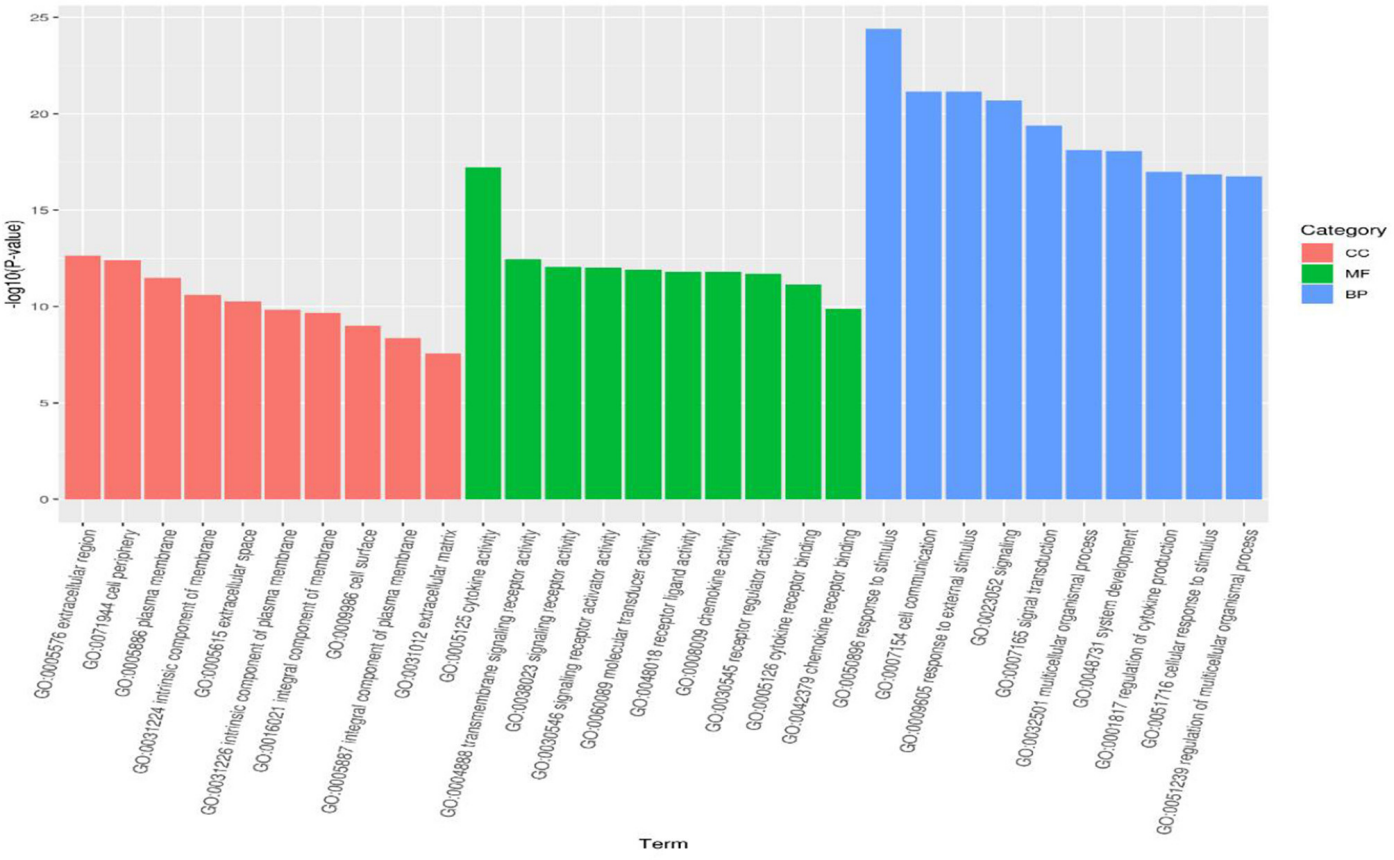
GO analysis for differentially expressed genes identified in the infected groups. The bar diagram displaying the top 10 GO terms of each category the x-axis shows significantly enriched GO terms, and the y-axis shows the -log_10_ *P*-value of these terms. The red, green and blue bars represent “Cellular Component” (CC), “Molecular Function” (MF) and “Biological Process” (BP) categories in GO, respectively.

To analyze the regulatory pathways and functions of the differentially expressed miRNAs in response to CDV infections, the intersectional differentially expressed genes (DEGs) of the infected groups compared with the negative control were subjected to gene function analysis. The functions in the identification of 7,675 GO terms included 6,000 terms in biological process, 582 terms in cellular component and 1,093 terms in molecular function. The top 10 GO enrichment items in each category are shown in a bar diagram (Figure 3). In terms of cellular components, GO-terms were mainly enriched in the extracellular region, cell periphery, plasma membrane and other processes. GO-terms in the biological process were enriched in response to stimulus, cell communication, external stimulus, signaling, multicellular organismal process and other processes. GO-terms in molecular function were enriched in cytokine activity, transmembrane signaling receptor activity, signaling receptor activity and others in molecular function.

**Figure 4:**
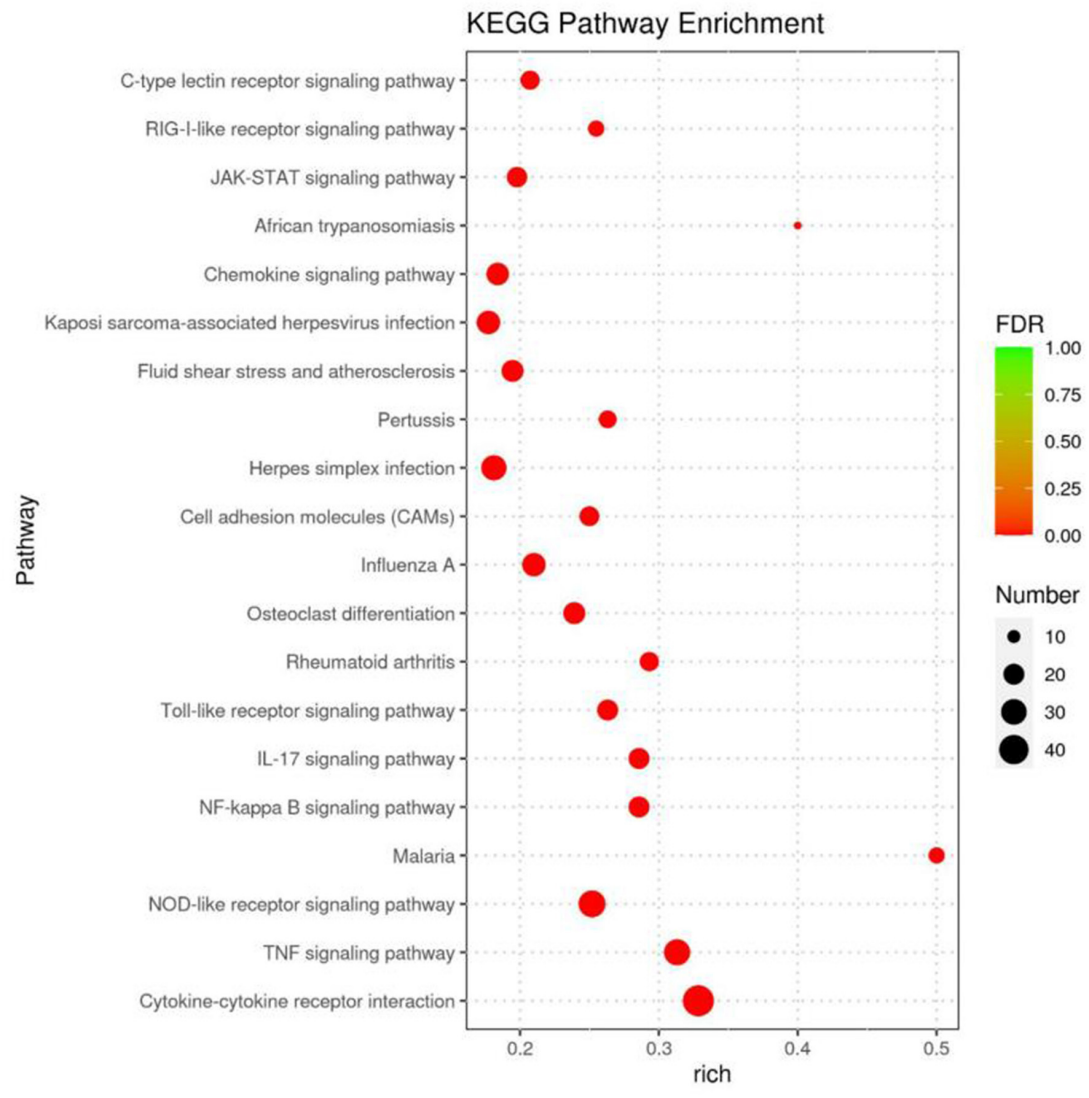
KEGG pathway analysis for the differentially expressed genes identified to the infected groups. The bubble diagram displays the top 20 items, the x-axis shows the score of enrichment, and the y-axis shows significantly enriched pathway terms. The size of each circle represents the number of genes enriched for the given pathway, where the larger the bubble, the more differentially expressed genes are contained.

A KEGG pathway enrichment analysis was performed, which identified 294 pathway terms. The top 20 terms are displayed in a bubble diagram (Figure 4). The items are associated with the infection of CDV, including the C-type lectin receptor signaling pathway, retinoic acid-inducible gene I (RIG-I)-like receptor signaling pathway, Janus kinase (JAK)-signal transducer and activator (STAT) signaling pathway, chemokine signaling pathway, toll-like receptor signaling pathway, interleukin 7 (IL-7) signaling pathway, nuclear factor (NF)-kappa B signaling pathway, nucleotide oligomerization domain (NOD)-like receptor signaling pathway, tumor necrosis factor (TNF) signaling pathway and cytokine-cytokine receptor interaction.

**Figure 5:**
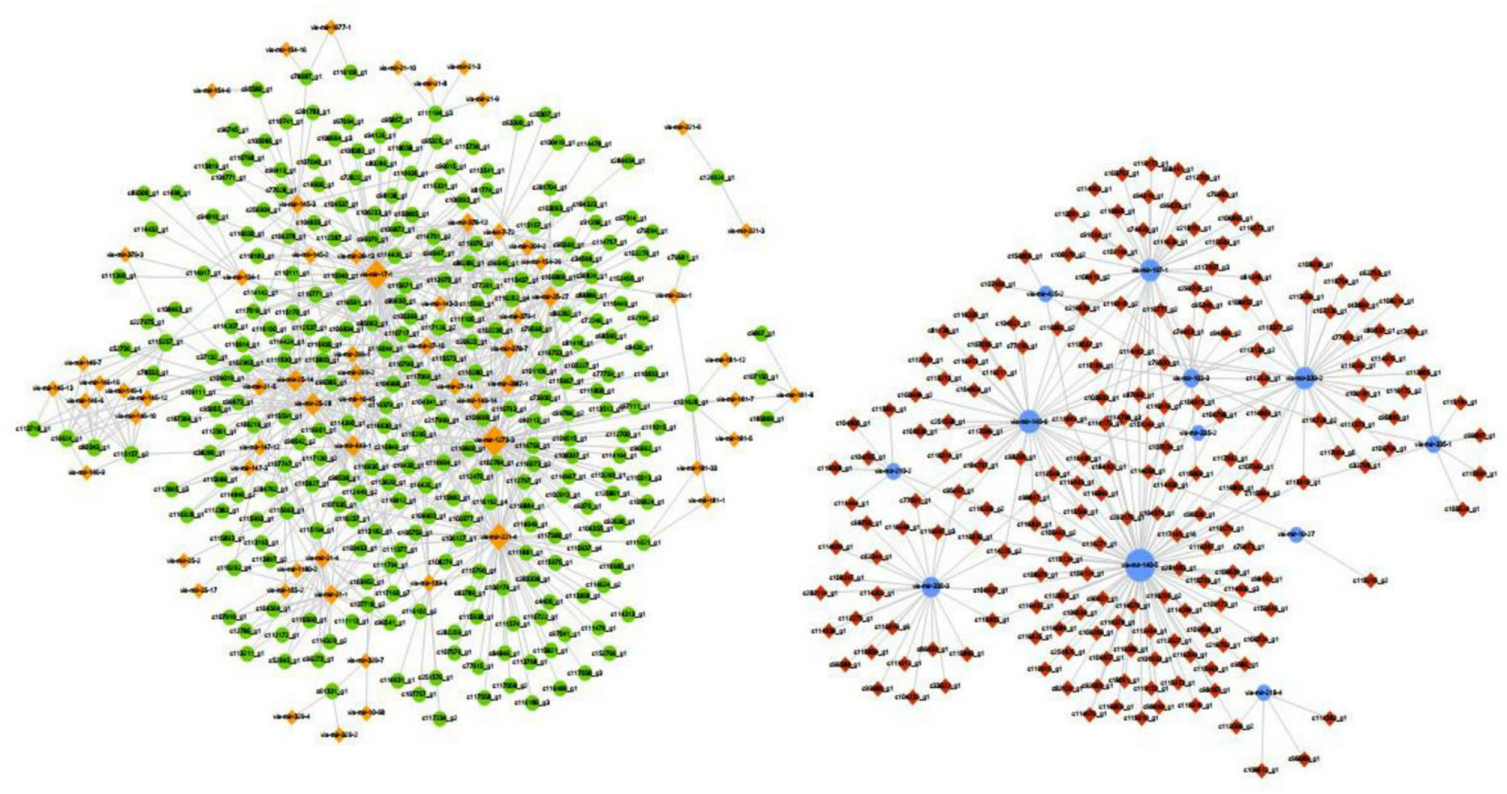
miRNA-mRNA interaction network. The blue circle represents miRNA downregulation, and yellow diamond represents miRNA upregulation. The red diamond represent target mRNA gene upregulation and green circle represent target mRNA gene downregulation.

Numerous miRNAs were observed to be involved in CDV infection, and an interaction network analysis of miRNAs and mRNAs was visualized using Cytoscape, which showed 555 nodes and 936 miRNA-mRNA pairs. The upregulated miRNA regulatory network contained 344 nodes and 674 pairs. The downregulated miRNA regulatory network consisted of 211 nodes and 262 pairs. The miRNA-mRNA interaction diagrams showed complex regulatory relationships that did not show simple one-to-one regulatory relationships between the miRNAs and mRNAs (Figure 5). According to the results of KEGG enrichment pathway and interaction network, miRNA and corresponding target genes of TNF signaling pathway, NOD-like receptor signaling pathway, interleukin 17 (IL-17) signaling pathway, toll-like receptor signaling pathway, RIG-I-like receptor signaling pathway, C-type lectin receptor signaling pathway, cytokine-cytokine receptor interaction, chemokine signaling pathway, JAK-STAT signaling pathway and NF-kappa B signaling pathway were showed in Table 1. Downregulation of mir-140-5p could cause upregulation of interleukin 27 Receptor Subunit Alpha (IL27RA), Vascular Cell Adhesion Molecule 1 (VCAM1), TNF Receptor Associated Factor 2 (TRAF2), G Protein-Coupled Receptor Kinase 1 (GRK1) and C-X-C Motif Chemokine Ligand 16 (CXCL16).

**Table 1:** Relationships between miRNAs and inversely correlated target genes.

**miRNA**	**Target gene**	**Pathway**
Upregulation	Downregulation	
mir-221-4	CCR1	Cytokine-cytokine receptor interaction, Chemokine signaling pathway
mir-139-4	THPO	Cytokine-cytokine receptor interaction, JAK-STAT signaling pathway
mir-1273-3	TNFRSF8	Cytokine-cytokine receptor interaction
mir-145-3	LEPR	Cytokine-cytokine receptor interaction, JAK-STAT signaling pathway
mir-17-1	MAPK12	TNF signaling pathway, NOD-like receptor signaling pathway, IL-17 signaling pathway, Toll-like receptor signaling pathway, RIG-I-like receptor signaling pathway, C-type lectin receptor signaling pathway
mir-204-2	IL-12	Cytokine-cytokine receptor interaction, JAK-STAT signaling pathway, Toll-like receptor signaling pathway, RIG-I-like receptor signaling pathway, C-type lectin receptor signaling pathway
mir-378-12	IL-1	Cytokine-cytokine receptor interaction, NF-kappa B signaling pathway
mir-181-8	TNFRSF11B	Cytokine-cytokine receptor interaction
Downregulation	Upregulation	
mir-425-2	PDGFB	JAK-STAT signaling pathway
mir-218-4	ADCY7	Chemokine signaling pathway
mir-140-6	LIF	Cytokine-cytokine receptor interaction, JAK-STAT signaling pathway, TNF signaling pathway
mir-330-3	CCL20	Cytokine-cytokine receptor interaction, Chemokine signaling pathway, TNF signaling pathway, IL-17 signaling pathway
mir-140-5p	GRK1	Chemokine signaling pathway
	IL-27	Cytokine-cytokine receptor interaction, JAK-STAT signaling pathway
	TRAF2	TNF signaling pathway, NOD-like receptor signaling pathway, IL-17 signaling pathway, NF-kappa B signaling pathway, RIG-I-like receptor signaling pathway
	CXCL16	Cytokine-cytokine receptor interaction, Chemokine signaling pathway
	VCAM1	TNF signaling pathway, NF-kappa B signaling pathway

## Discussion

Up to date, the latest release of the miRBase database (v22) contains 38,589 entries representing hairpin precursor microRNAs. Those hairpin precursors produce a total of 48,860 different mature microRNA sequences (27). As a class of non-coding RNAs, miRNAs regulate the immune, metabolic and growth processes of living organisms by inhibiting the expression of their target genes. In recent years, high-throughput sequencing techniques were used to determine the expression profiles of miRNAs in animals, plants and viruses. Some studies showed that the integrated analysis of miRNA and mRNA in viral infection samples could confirm the regulatory mechanism of miRNA (28–30). The analysis of giant pandas in captivity vaccinated against CDV was operated with miRNA-mRNA. It revealed that downregulation of mir-204 might increase toll-like receptor 6 (TLR6) expression and enhance innate immunity of giant panda cubs, and downregulation of mir-330 might activate macrophages and regulate immune response by increasing transmembrane protein 106A (TMEM106A) expression (31). Twenty miRNAs (6 upregulated; 14 downregulated) and 1,286 mRNAs (935 upregulated; 351 downregulated) showed the same differential expression trend in influenza A virus infected A549 cells in the three groups as in the uninfected control group (32). In this study, the integrated analysis of miRNA-mRNA expression in Mv. l. Lu cells infected with CDV was performed for the first time. Mv. l. Lu cells were selected for this study because they could support CDV replication *in vitro* and were homologous cells of minks. The analysis of mRNA and miRNA showed that 4,734 differentially expressed mRNAs (2,691 upregulated and 2,043 downregulated) and 181 differentially expressed miRNAs (152 upregulated and 29 downregulated) were detected, respectively. KEGG pathway enrichment of the combination of miRNA and mRNA was done for analyzing immune and inflammatory responses. The relationships between miRNAs and target genes suggested that upregulation and downregulation of miRNA might influence JAK-STAT signaling pathway, NF-kappa B signaling pathway, TNF signaling pathway, NOD-like receptor signaling pathway, interleukin 17 (IL-17) signaling pathway, toll-like receptor signaling pathway, RIG-I-like receptor signaling pathway, C-type lectin receptor signaling pathway, cytokine-cytokine receptor interaction and chemokine signaling pathway.

JAK-STAT signaling pathway and NF-kappa B signaling pathway were found in gene enrichment analysis. JAK-STAT signaling pathway is a cytokine-stimulated signal transduction pathway, which is involved in many important biological processes, such as cell proliferation, differentiation and apoptosis, and immune regulation. Many cytokines and growth factors provide signals through the JAK-STAT signaling pathway, including interleukin, GM-CSF (granulocyte/macrophage colony-stimulating factor), GH (growth hormone), EGF (epidermal growth factor), PDGF (platelet-derived factor), IFN (interferon), and so on. In the JAK-STAT pathway, JAK phosphorylates STAT to form a dimer and directly regulates the expression levels of related genes in the nucleus, thus playing an important role in the body's immune response (33). Signal transducer and activator of transcription 1 (STAT1), signal transducer and activator of transcription 2 (STAT2) and signal transducer and activator of transcription (STAT3) phosphorylation upregulated IFN-stimulating gene (ISG) transcription and improved cell's antiviral ability (34). In our study, the expression levels of JAK-STAT signaling pathway-related factors changed significantly, such as upregulation of platelet derived growth factor subunit B (PDGFB), interleukin 6 family cytokine (LIF), interleukin 27 (IL-27) and downregulation of thrombopoietin (THPO), leptin receptor (LEPR) and interleukin 12 (IL-12). Preliminary results suggested that the JAK-STAT signaling pathway might be activated in Mv. l. Lu cells infected with CDV. PDGFB and LIF were strongly associated with STAT3 activation (35, 36). THPO-stimulated STAT3 and signal transducer and activator of transcription (STAT5) expression can potentiate MPL signaling through the JAK-STAT signaling pathway (37). THPO leads to S6 phosphorylation over signaling to STAT5 (38). LEPR is a pleiotropic molecule, which appears in a variety of tissues, affects certain cellular functions and acts as an inflammatory cytokine. The high expression of mir-153 can inhibit activation of the JAK-STAT signaling pathway by LEPR (39). Cytokines, such as IL-12 and IL-27 *via* JAK-STAT signaling activate immune reactions.

NF-kappa B can be expressed in all cell types and regulates the expression levels of a large number of immune cytokines, such as interleukin 2 (IL-2), intercellular adhesion molecules (ICAM-1) and vascular endothelial growth factor (VEGF). It is involved in most intracellular immune responses. In the process of the natural immune response, the most important function of NF-kappa B is to participate in the inflammatory response. NF-kappa B activation pathways are divided into classical and non-classical activation pathways. The classical activation pathway is activated by most proinflammatory factors, such as TNF, interleukin 1 (IL-1), or viral and bacterial products. Several viral infections, including porcine parvovirus (PPV), herpes simplex virus (HSV) and measles virus (MV), were reported to activate NF-kappa B signaling pathway (40–42). Epstein-barr virus (EBV) could activate NF-kappa B signaling pathway and inhibit viral replication (43). In PCV2 infected PK15 cells, NF-kappa B is activated concomitantly with viral replication characterized by cell death (44). In this study, the activation of the NF-kappa B signaling pathway in Mv. l. Lu cells infected with CDV was determined through the upregulated expression of TRAF2 and VCAM1 and downregulated expression of IL-1. TRAF family members are a large group of intracellular junction proteins that can directly or indirectly bind to various TNF and IL-1/TLR family members. They mediate the signaling of a variety of downstream signaling pathways, including the NF-kappa B signaling pathway. It affects cell survival, proliferation, differentiation and death and regulates many biological processes. In NF-kappa B signaling pathway, TRAF2 plays an important role in the activation of the IkappaB kinase (IKK) complex. NF-kappa B can significantly promote tumor metastasis and the expression of tumor metastasis-related gene VCAM-1.

The enrichment analysis of target mRNAs of differentially expressed miRNAs revealed that mir-140-5p and mir-378-12 targeted corresponding genes to regulate NF-kappa B signaling pathway. JAK-STAT signaling pathway could be modulated by mir-425-2, mir-139-4, mir-140-6, mir-145-3, mir-140-5p and mir-204-2. mir-425, mir-139, mir-145, mir-378, mir-204 and mir-140 play their regulatory roles by acting on different signaling pathways in the body (45–50). Downregulation of mir-140-5p caused upregulation of IL-27, TRAF2, VCAM1, GRK1 and CXCL16 and modulated JAK-STAT signaling pathway, NF-kappa B signaling pathway, chemokine signaling pathway, cytokine-cytokine receptor interaction, TNF signaling pathway, NOD-like receptor signaling pathway, IL-17 signaling pathway and RIG-I-like receptor signaling pathway. Our results suggest that mir-140-5p affected various signaling pathway in Mv. l. Lu cells infected with CDV. There were some studies of mir-140-5p were reported, such as mir-140-5p directly targeted Nrf2 and Sirt2 and thereby increasing DOX-caused myocardial oxidative damage, mir-140-5p negatively regulated TLR4 to mediate inflammation in 16HBE cells and mir-140-5p suppressed activation of the Wnt/β-catenin and NF-κB pathways by targeting SOX4. Thus, mir-140-5p could affect some signaling pathways through target genes (51–53). This study preliminarily analyzed the correlation of miRNA and mRNA after CDV infected Mv. l. Lu cells, but the live experimental animals infected with CDV *in vivo* would better clarify the expression levels of miRNAs and mRNAs. This study supported in prospective studies with *in vivo* analyses using live experimental animals.

## Conclusion

This study analyzed the expression levels of miRNAs and mRNAs in CDV infected Mv. l. Lu cells. We identified 4,734 differentially expressed mRNAs and 181 differentially expressed miRNAs. The DEGs were associated with various biological processes and signaling pathways, such as JAK-STAT signaling pathway, NF-kappa B signaling pathway and other immune-related pathways. The target genes of mir-140-5p modulated various signaling pathways. This study revealed preliminaryly that downregulation of mir-140-5p may play an important role in the immune response of Mv. l. Lu cell infected with CDV, the specific mechanism needs further study.

## Data Availability Statement

The datasets presented in this study can be found in online repositories. The names of the repository/repositories and accession number(s) can be found in the article/supplementary material.

## Author Contributions

QC, NS, SC, and QZ designed the study. QC, MT, and YY performed the experiments. LY, GW, and SC analyzed the data. ZC and YC prepared the figures and tables. QC wrote the manuscript. All authors have read and approved the manuscript.

## Funding

This study was funded by the Jilin Province Scientific and Technological Program (20190301086NY) and Jilin Province Science and Technology Development Plan Project (20200402036NC), the Central Guidance on Local Science and Technology Development Fund of Jilin Provincial Research Foundation for basic Research (202002058JC).

## Conflict of Interest

The authors declare that the research was conducted in the absence of any commercial or financial relationships that could be construed as a potential conflict of interest.

## Publisher's Note

All claims expressed in this article are solely those of the authors and do not necessarily represent those of their affiliated organizations, or those of the publisher, the editors and the reviewers. Any product that may be evaluated in this article, or claim that may be made by its manufacturer, is not guaranteed or endorsed by the publisher.
